# Examining the cost burden of dietary supplements in older adults: an analysis from the AAA longroad study

**DOI:** 10.1186/s12877-025-05823-x

**Published:** 2025-03-15

**Authors:** Sara Baird, Ryan Moran, Sarah Hacker, Dylan Lawton, Linda Hill

**Affiliations:** 1 Herbert Wertheim School of Public Health and Human Longevity Science, University of California San Diego, San Diego, CA USA; 2 University of Hawai’i John A. Burns School of Medicine, Honolulu, HI USA

**Keywords:** Older adult, Dietary supplement, Supplement, Polypharmacy, Medication cost

## Abstract

**Background:**

The use of dietary supplements (DS) has steadily increased over the last several decades, particularly among older adults, contributing to the growth of the multibillion-dollar DS industry. The cost of prescription medication is a known contributor to medication nonadherence, yet the cost burden of DS among older adults is not well understood.

**Methods:**

Using medication data from the 5-year multicenter longitudinal cohort AAA LongROAD study of older adults who drive, DS were identified and categorized. Cost estimates were based on prices obtained from a popular online marketplace, using dosing and frequency recommendations from the National Institutes of Health Office of Dietary Supplements database. ANOVA was used to explore associations between demographics and DS cost burden.

**Results:**

Of the 2,990 participants at baseline, 2068 (69%) followed up through year 5. The number of DS users ranged from 70.4 to 82.7% of the participants from baseline to year 5. Among the 160 supplement formulations identified, 142 (88%) had price data and were included in the analysis. The mean estimated cost of individual supplements ranged from $0.73 to $49.59 per month. The mean monthly cost burden for all older adult participants ranged from $10.23 (SD 14.74) at baseline to $13.14 (SD 16.93) in year 3, with a mean annual cost burden of $142 per participant across all years. The mean monthly cost burden for DS users only ranged from $14.56 (SD 15.59) at baseline, to $16.45 (SD 17.45) in year 3, with a mean annual cost burden of $186 per DS user across all years. Increased spending was associated with female gender, older age, higher income, not working, and being White non-Hispanic.

**Conclusion:**

The use of DS is common among older adults. Using conservative estimates of monthly cost, the spending of older adults on DS is high. The real-world impact of DS costs on older adults, such as the impact on the affordability of prescription medication, is a key point for future research.

**Clinical trial number:**

Not applicable.

**Trial registration:**

Not applicable.

## Background

Dietary supplements (DS) include a heterogeneous grouping of vitamins, herbal and botanical compounds, amino acids, minerals, and probiotics [1]. While patients report taking DS to improve overall health or maintain health, in addition to specific interests such as cardiovascular disease [1, 2], consistent evidence of benefit and effectiveness remains lacking [1, 3], and the use of DS is not without risk [4].

Despite this, the use of DS is widespread and has increased in the last several decades in the United States [5], particularly among older adults [6]. The National Health and Nutrition Examination Survey from 2017 to 18 reported that 74% of U.S. adults over age 60 used some form of DS in the prior 30 days [7], whereas other surveys in middle aged and older adult populations reported regular use rates above 80% [8, 9].

Many older adults have fixed incomes [10], and while considerable attention has been given to the cost burden of prescription pharmacologic agents on older adults [11], few data exist concerning the cost burden of DS. Recent analysis suggested that more than 3 million older adults on Medicare are unable to afford prescription medications, and survey data from Americans 50 and older suggest that a sizable minority do not fill prescribed medications, with cost being the most cited reason [12]. These findings were echoed in another pool of older adults over age 65, in which over 1 in 5 reported not filling or taking medications because of cost, and almost a quarter reported difficulty affording prescription medication [13]. Despite these understandable cost-conscious behaviors, in 2022, the global DS market was valued at $169 billion and is predicted to increase to $330 billion in 2030 [14]. As most insurance payers, including Medicare do not cover DS, individuals often pay for DS out of pocket [15].

While data surrounding consumer patterns note growth in DS spending, the actual cost burden of DS among older adults in real-world settings is unclear. Furthermore, as a population, older adults are increasingly utilizing DS while concurrently struggling to afford prescription medications. Therefore, there is a need to better quantify the cost burden of DS in older adults. The purpose of the current analysis was to assess the DS cost burden in older adults using data from the AAA Longitudinal Research on Aging Drivers (LongROAD) study.

## Methods

The LongROAD study was a multicenter, prospective cohort study of older adults (≥ 65 years old) who drive, and was designed to understand the medical, behavioral, medical, environmental, and vehicle technologic factors that influence driving behavior. The methods of the LongROAD study are published elsewhere [16], but briefly and relevant to this analysis, older adults were invited to participate across five study locations (Ann Arbor MI, Denver CO, Cooperstown NY, Baltimore MD, and San Diego CA) from July 2015 to March 2017 and were followed for 5 years. At baseline and annually, all participants underwent a detailed medication review either by phone or by in-person ‘brown bag’ review, where participants brought in all prescribed and OTC medications, including all supplements. Medications were reviewed in real time with participants by study personnel and entered into a database. Study protocol called for in person review at baseline, year 2, and year 4, and telephone review at years 1, 3 and 5. However, due to the COVID-19 pandemic, some participants were unable to perform a year 4 review in person and instead did a telephone review; these were followed by an in-person review in year 5. All participants had a total of three in-person and three telephone-collected medication reviews throughout the 5-year study period. The baseline demographics used in this subanalysis were collected in person at each study site.

Institutional Review Board approval was obtained at each study site, and written informed consent was obtained from all participants.

### DS identification and grouping

Methods for the categorization of medications and identification and grouping of supplements have been published elsewhere [16, 17]; however, briefly and pertinent to this analysis, prescription and OTC medications, including DS, were systematically coded on the basis of the American Hospital Foundation Service (AHFS) system [18]. The AHFS classification allows the grouping of drugs with similar pharmacologic, therapeutic and/or chemical characteristics in a four-tier hierarchy. While the AHFS system allows for the classification of some vitamins and minerals, other medications that could not be classified included food-like items (e.g., turmeric), homeopathic compounds, and other supplements such as saw palmetto. The final release of data including all five cohort years underwent centralized refinement and is presented in this paper.

Dietary supplements were identified from the LongROAD medication database by reviewing non-classified medications as well as classified medications from the AHFS Tier 88 (Vitamins) and Tier 40:12 (Electrolyte, Caloric, and Water Balance - Replacement Preparations). Medications in these tiers that were prescribed with clear pharmacologic purposes were excluded from being included as a DS. This DS database was then reviewed, and supplements were grouped based on their listed components. The groupings included individual supplements, multivitamins, B combinations, eye vitamins, artificial tears, topicals, probiotics, digestive enzymes, elemental minerals, cannabis products, amino acids, other, and undetermined. Medications were re-reviewed with minor corrections for analysis of the full 5-year dataset. Three DS groupings were excluded from the cost analysis because they either had heterogeneous components (other, topical) or not enough information (undetermined).

### Cost estimates

Cost estimates were collected by finding the lowest priced available formulation in a popular online marketplace during the summer of 2022 and applied to the study period. Specific DS categories were searched and filtered to show results from lowest to highest. The results were manually reviewed to confirm the ingredient list and appropriateness for inclusion. When possible, DS were selected that matched items registered in the National Institutes of Health Office of Dietary Supplements (NIH ODS) database [19].

The use of an online marketplace was intended to provide a conservative (low) estimate of the cost burden. To confirm this, cost comparisons were made by auditing online prices for four nationwide retail pharmacies. All four pharmacies have in-person stores allowing in-store pick-up or home delivery; one organization is membership-based. Prices were also confirmed by in-person visits to 2 brick-and-mortar locations of one retailer in different geographic locations (Washington DC, San Diego CA). Audits consisted of finding the lowest price DS for the top 10 combined DS formulations (multivitamins, vitamin D, omega 3, calcium/vitamin D combination, vitamin C, Vitamin B12, eye vitamin formulations, coenzyme Q10, and magnesium).

The cost burden per participant was calculated based on the frequency of use as suggested by the NIH ODS and the cost per pill for each DS reported. When ODS data were unavailable, the physician researchers determined the recommended dosing in conjunction with label instructions. Daily cost estimates were used to create monthly (30-day) cost estimates. Calculations assumed continuous use for one year until the next medication review period. Cost calculations across the cohort were performed for both all participants to estimate costs in older adult populations, as well as for only DS users to help distinguish the average cost burden among those who utilized DS.

### Statistical analysis

ANOVA was used to explore associations between demographics and propensity to pay more for DS compounds, with *p* < 0.05 indicating statistical significance, and with the use of Bonferroni adjustments for multiple comparisons. The mean monthly cost of DS and differences in cost by demographic group were adjusted for all other listed demographic variables, with *p* < 0.05 indicating statistical significance and with Bonferroni adjustments for multiple comparisons. All analyses were conducted using SAS v. 9.4 (SAS Institute; Cary, NC) and SPSS v. 28 (IBM; Armonk, NY).

## Results

A total of 2,990 participants were enrolled and completed the baseline review, and 2068 (69.2%) participants had medication and/or demographic information collected through year 5. Of the 79,726 individual entries in the DS database, 160 DS formulations were identified. The top ten DS formulations over 5 years were consistent with the preliminary 2-year findings [17], see Table 1. The top 10 formulations represented 66.4% of the total DS burden over the 5 years. The mean number of DS used per DS user ranged from a low of 3.34 at baseline, to a high of 3.80 in year 5, with an overall mean of 3.54 DS per DS user per year over the study period. For all participants, the mean number of DS per participant ranged from a low of 2.35 at baseline to a high of 3.14 in year 5, with a mean of 2.69 DS per participant per year over the study period.

Among the 160 DS formulations identified, 142 (88.7%) were included for cost analysis; most of the remainder either had no available cost data (*N* = 15) or had their grouping excluded (*N* = 3; Topical, Other, Undetermined). Among the 142 included DS, 73 (51.4%) were referenced in the ODS, and ODS guidelines were used.

### Cost comparison

For the pharmacy audits of the top 10 DS at four retail pharmacies online, comparison prices were available for 38 of the 40 items queried. The overall mean cost of the study’s selected online retailer was 87% of the mean cost of the retail pharmacies online (range 46—126%). For the prices of the top ten DS, when the study retailer was compared with the four comparison pharmacies, the mean cost was greater for two DS (Omega 3, Calcium/Vitamin D), whereas the remaining eight of the top DS had lower mean prices at the study retailer. The two in-store retail pharmacies had slightly higher mean costs than their online equivalents.

### Cost of supplement formulations

The most and least expensive DS were Omega 3 + Turmeric ($49.30/month) and Zinc ($0.73/month). The DS cost distribution was positively skewed, with a mean DS cost of $9.65/month and a median of $5.50/month. The 75th percentile of the DS cost was $11.65/month.

**Table 1:** Estimated cost of the top 10 most common DS formulations taken by the AAA longroad cohort of older adults who drive

	Estimated monthly cost	% Participants*
Multivitamin	$1.30	39.4%
Vitamin D	$0.93	39.1%
Omega 3	$12.34	20.6%
Calcium + Vitamin D	$5.49	10.8%
Vitamin C	$1.22	10.6%
Calcium	$2.17	10.2%
Vitamin B12	$1.62	10.1%
Coenzyme Q	$19.02	8.7%
Eye Vitamin	$12.05	7.7%
Magnesium	$1.65	7.6%

* Measured across all time points

### Mean monthly cost burden

Among all participants, the estimated mean monthly cost of all DS ranged from $10.23 (SD 14.74) at baseline, to $13.32 (SD 16.56) in year 5, corresponding to an estimated mean annual cost range of $122.76 at baseline to $159.84 in year 5, and a mean annual cost of $141.68 over all time periods ($11.81 per month). Among DS users, the estimated mean monthly cost of DS ranged from $14.56 (SD 15.59) at baseline, to $16.45 (SD 17.45) in year 3, corresponding to an estimated mean annual cost range of $174.72 to $197.40 and a mean annual cost of $186.26 over all time periods ($15.52 per month). The minimum monthly estimated cost was $0.73, and the maximum monthly estimated cost was $174.80. Further detail is noted in Table 2.

**Table 2:** Mean monthly cost of dietary supplements over time, for DS users only and all participants

	Baseline	Year 1	Year 2	Year 3	Year 4	Year 5
**All Participants**						
N	2990	2869	2714	2551	2226	2068
Mean Cost	$10.23	$12.16	$11.00	$13.14	$10.99	$13.32
SD Cost	$14.74	$15.82	$15.80	$16.93	$14.78	$16.56
**DS Users**						
N	2105	2233	1940	2040	1692	1711
Mean Cost	$14.56	$15.64	$15.42	$16.45	$14.94	$16.12
SD Cost	$15.59	$16.35	$16.79	$17.45	$15.40	$16.93
**Minimum Cost+**	$0.73	$0.91	$0.91	$0.91	$0.73	$0.91
**Maximum Cost**	$130.00	$138.25	$174.80	$174.80	$104.67	$117.03

+Minimum cost for DS users. Minimum mean cost for all participants was $0.00, as some participants did not use DS

### Cost burden by demographics

The results of the multivariate analysis of the baseline demographics and the monthly mean cost for all participants are presented in Table 3. Between 70.5% (baseline) and 82.7% (year 5) of participants took at least one DS during each study year.

After adjusting for other demographic variables, participants from Baltimore spent less than participants from Denver in all years, less than those from New York in year 3, and less than those from San Diego in years 2, 3 and 4. Participants in the youngest age group (65–69 years) tended to spend less per month than those in the older age groups, though this difference varied by study year. Females spent more for DS than men at all study time points. White non-Hispanic participants spent significantly more than Black non-Hispanic participants at all time points. There were no other significant differences between race/ethnicity except in year 4, when Asian participants spent more than Black non-Hispanic participants. During most study years, those who reported working at baseline spent more than those who did not. There were no significant differences in DS monthly costs based on education or income.

**Table 3:** Demographics and mean monthly DS cost of the older adult participants of the AAA longroad study from baseline to year 5

Cost of Dietary Supplements						
	Baseline	Year 1	Year 2	Year 3	Year 4	Year 5
	DS users/total Mean monthly cost+ (SD)	*N* mean (SD)	*N* mean (SD)	*N* mean (SD)	*N* mean (SD)	*N* mean (SD)
**Total Participants**	2990	2869	2714	2551	2226	2068
**% of DS Users**	70.4%	77.8%	71.5%	80.0%	76.0%	82.7%
**Study Site**						
Denver, CO (A)	466/600 11.94 (16.53) ^c, e^	466/559 14.74 (17.61) ^b, c,e^	362/524 12.18 (17.13) ^c^	417/515 15.57 (18.66) ^c, d^	374/476 12.99 (15.36) ^c^	390/460 15.65 (17.38) ^c^
Cooperstown, NY (B)	428/601 10.39 (13.85) ^e^	436/584 11.34 (14.42)	422/560 11.90 (16.21) ^c^	384/500 13.31 (17.48) ^c^	332/433 11.21 (13.86) ^c^	329/409 12.85 (15.66) ^c^
Baltimore, MD (C)	428/588 9.25 (13.70)	436/559 11.00 (14.79)	326/529 7.69 (13.10)	392/513 9.98 (14.65)	307/467 7.83 (13.13)	286/390 9.52 (15.05)
Ann Arbor, MI (D)	472/601 11.95 (14.73) ^c, e^	480/592 12.83 (15.94)	425/564 11.19 (14.93) ^c^	409/513 12.52 (15.62)	323/387 11.49 (15.42) ^c^	315/360 13.88 (17.16) ^c^
San Diego, CA (E)	314/600 7.60 (14.29)	404/575 10.92 (15.90)	392/537 11.98 (16.91) ^c^	425/510 14.36 (17.50) ^c^	343/463 11.46 (15.56) ^c^	383/449 14.23 (16.73) ^c^
**Age group**						
65–69 years (A)	848/1243 8.98 (13.12)	688/910 10.67 (14.16)	437/641 8.85 (13.06)	305/398 10.27 (14.77)	121/164 8.84 (11.64)	0 - (-)
70–74 years (B)	710/1037 10.22 (14.90)	844/1100 11.93 (15.84)	772/1103 11.34 (16.04) ^a^	843/1080 13.11 (16.32) ^a^	748/1019 10.06 (14.11)	734/911 11.82 (15.09)
75–79 years (C)	530/710 12.43 (16.82) ^a, b^	604/753 13.65 (16.89) ^a^	549/753 11.78 (16.61) ^a^	615/755 14.00 (16.97) ^a^	524/680 11.49 (14.85)	601/725 13.61 (16.46)
80 and over (D)	0 - (-)	81/100 17.46 (19.53) ^a, b^	169/216 13.02 (17.12)	261,315 14.86 (18.25) ^a^	286/363 13.64 (15.17) ^a, b^	367/430 16.08 (17.19) ^b, c^
**Gender**						
Male (A)	895/1403 8.55 (13.49)	948/1340 10.06 (14.22)	811/1266 8.95 (13.86)	872/1197 10.74 (14.96)	712/1027 9.29 (14.12)	725/950 11.30 (15.55)
Female (B)	1193/1587 11.70 (15.62) ^a^	1269/1523 14.02 (16.90) ^a^	1116/1447 12.79 (17.12) ^a^	1152/1351 15.28 (18.25) ^a^	967/1199 12.44 (15.17) ^a^	977/1116 15.07 (17.19) ^a^
**Race/Ethnicity**						
White, Non-Hispanic (A)	1813/2559 10.63 (14.92) ^b^	1911/2453 12.55 (16.04) ^b^	1681/2327 11.58 (16.25) ^b^	1742/2187 13.60 (17.21) ^b^	1468/1914 11.35 (14.82) b	1483/1782 13.80 (16.85) ^b^
Black, Non-Hispanic (B)	134/213 6.46 (12.97)	146/203 7.71 (13.35)	111/191 5.38 (9.70)	130/178 7.12 (12.54)	91/155 5.24 (11.05)	95/136 7.54 (12.13)
American Indian (C)	13/18 9.26 (12.94)	14/18 12.44 (17.37)	11/16 11.74 (18.13)	9/13 16.04 (20.52)	10/11 15.09 (20.64)	11/11 13.37 (19.14)
Asian (D)	35/64 10.64 (16.25)	43/60 13.44 (17.47)	44/59 10.70 (13.70)	50/56 15.21 (15.37) b	34/47 11.70 (16.50)	35/43 13.16 (16.72)
Alaska Native, Native Hawaiian, Pacific Islander (E)	6/9 6.54 (6.15)	7/9 7.52 (6.96)	6/8 6.20 (11.33)	7/7 9.77 (12.18)	5/6 8.74 (12.87)	6/6 13.25 (18.68)
Other, Non-Hispanic (F)	21/32 10.57 (14.59)	25/31 12.97 (14.28)	17/28 10.00 (17.64)	22/28 14.81 (19.17)	19/26 13.76 (18.94)	20/24 16.50 (18.18)
Hispanic (G)	55/83 7.51 (12.53)	60/77 10.90 (13.62)	48/72 8.54 (11.85)	55/68 12.20 (15.86)	43/57 12.07 (15.05)	45/56 11.51 (12.80)
Refused (H)	11/12 10.08 (9.83)	11/12 12.45 (13.41)	9/12 10.97 (15.71)	9/11 11.73 (14.03)	9/10 10.79 (13.90)	7/8 10.99 (15.47)
**Education**						
HS deg. or less (A)	226/336 9.89 (14.93)	208/313 11.19 (15.63)	208/294 10.82 (16.23)	202/260 12.53 (17.16)	158/222 10.12 (15.82)	149/186 13.30 (18.84)
Some college/ Vocational (B)	374/528 9.39 (13.86)	512 11.07 (15.22)	324/478 9.83 (15.13)	335/442 11.67 (17.04)	280/380 10.25 (15.51)	281/340 12.74 (17.68)
Associate’s/Bachelor’s deg. (C)	623/896 10.21 (14.33)	863 12.38 (15.80)	583/817 11.74 (16.17)	611/775 13.59 (16.84)	493/663 11.09 (14.41)	522/637 13.60 (16.61)
Advanced college deg. (D)	861/1221 10.72 (15.35)	1167 12.79 (16.15)	808/1116 11.05 (15.71)	874/1065 13.64 (16.92)	746/957 11.42 (14.50)	746/897 13.42 (15.60)
**Household income**						
Less than $20,000 (A)	81/134 6.78 (10.39)	86/123 8.44 (15.48)	71/108 6.98 (10.95)	77/108 9.35 (12.31)	56/85 7.79 (13.34)	64/85 9.97 (13.88)
$20,000 - $49,999 (B)	468/640 10.57 (14.29)	523/651 12.04 (14.82)	458/632 11.21 (16.56)	471/578 13.59 (17.61)	373/493 10.72 (14.12)	357/438 12.58 (16.49)
$50,000 - $79,999 (C)	512/720 11.25 (16.11) ^a^	550/701 13.34 (17.19) ^a^	462/667 11.22 (16.48)	502/635 13.71 (18.13)	423/552 11.64 (15.48)	428/509 14.14 (16.82)
$80,000 - $99,999 (D)	307/431 10.27 (14.42)	318/407 13.11 (16.47) ^a^	288/381 11.57 (15.63)	284/349 13.59 (16.62)	249/313 11.82 (15.25)	251/294 14.48 (17.51)
$100,000 or greater (E)	638/959 9.48 (14.51)	668/897 11.26 (15.16)	593/853 10.67 (15.01)	644/821 12.76 (16.11)	537/735 10.51 (14.65)	567/700 12.96 (16.22)
**Work status^**						
No (A)	1472/2084 10.66 (14.90) ^b^	1618/2067 12.61 (16.12) ^b^	1471/2010 11.51 (15.98) ^b^	1580/1963 13.42 (17.02)	1364/1778 11.27 (14.74) ^b^	1422/1704 13.59 (16.62)
Yes (B)	614/904 9.19 (14.32)	597/793 11.08 (15.03)	451/694 9.62 (15.26)	442/581 12.29 (16.69)	311/441 9.71 (14.38)	279/360 12.15 (16.29)

a-h Superscripts reflect significance (*p* < 0.05) compared to the associated subgroup (A-H)

*: significant *p* < 0.05 against all other categories combined, with Bonferroni adjustment for multiple comparisons

^: “Did you work for pay at any time in the past month?”

+: Mean cost for all participants (DS users and non-users)

### Analysis of covariance

The mean monthly cost across all years by demographics is presented in Table 4. After adjusting for other listed demographic variables, the site in Denver CO was associated with increased spending compared with all other sites (*p* < 0.001), whereas Baltimore MD was associated with lower spending (*p* < 0.001). Black non-Hispanic participants spent significantly less than all other racial/ethnic groups. Participants with a college degree or above spent significantly more than those with high school or some college education. The participants in the lowest income bracket (< 20,000 annual household income) had a lower estimated monthly DS cost burden than those in all other income groups (*p* < 0.001). Those who reported not working at baseline had a higher estimated monthly DS cost burden than those who reported working (*p* = 0.025) (Table 4).

**Table 4:** Mean estimated monthly cost of DS and differences in cost by baseline demographic groups, adjusting for other variables. All participants, all years

	Mean monthly cost ($)	Difference in mean monthly cost between groups (*p*-value)							
**Study Site**		A	B	C	D	E			
Denver, CO (A)	14.02		2.33 (< 0.001)	4.31 (< 0.001)	2.05 (< 0.001)	2.92 (< 0.001)			
Cooperstown, NY (B)	11.69			1.99 (< 0.001)	-0.28 (1.00)	0.60 (1.00)			
Baltimore, MD (C)	9.70				-2.27 (< 0.001)	-1.39 (0.007)			
Ann Arbor, MI (D)	11.97					0.87 (0.334)			
San Diego, CA (E)	11.10								
**Age group**		A	B	C	D				
65–69 years (A)	9.39		-1.99 (< 0.001)	-3.45 (< 0.001)	-5.97 (< 0.001)				
70–74 years (B)	11.38			-1.46 (< 0.001)	-3.68 (< 0.001)				
75–79 years (C)	12.84				-2.22 (< 0.001)				
80 and over (D)	15.06								
**Gender**		A	B						
Male (A)	9.60		-3.96 (< 0.001)						
Female (B)	13.55								
**Race/Ethnicity**		A	B	C	D	E	F	G	H
White, Non-Hispanic (A)	12.09		5.23 (< 0.001)	-0.51 (1.00)	-1.10 (1.00)	3.15 (1.00)	-1.46 (1.00)	2.03 (0.292)	0.567 (1.00)
Black, Non-Hispanic (B)	6.86			-5.74 (0.031)	-6.33 (< 0.001)	-2.08 (1.00)	-6.69 (< 0.001)	-3.20 (0.013)	-4.66 (0.845)
American Indian (C)	12.60				-0.59 (1.00)	3.66 (1.00)	-0.95 (1.00)	2.54 (1.00)	1.08 (1.00)
Asian (D)	13.19					4.25 (1.00)	-0.37 (1.00)	3.13 (0.22)	1.67 (1.00)
Alaska Native, Native Hawaiian, Pacific Islander (E)	8.95						-4.61 (1.00)	-1.12 (1.00)	-2.58 (1.00)
Other, Non-Hispanic (F)	12.56							3.49 (0.415)	2.03 (1.00)
Hispanic (G)	10.07								-1.461 (1.00)
Refused (H)	11.53								
**Education**		A	B	C	D				
HS deg. or less (A)	10.66		0.08 (1.00)	-1.37 (0.02)	-1.53 (0.007)				
Some college/ Vocational (B)	10.58			-1.46 (0.001)	-1.61 (< 0.001)				
Associate’s/Bachelor’s deg. (C)	12.03				-0.15 (1.00)				
Advanced college deg. (D)	12.19								
**Household income**		A	B	C	D	E			
Less than $20,000 (A)	7.90		-3.55 (< 0.001)	-4.53 (< 0.001)	-4.56 (< 0.001)	-3.56 (< 0.001)			
$20,000 - $49,999 (B)	11.45			-0.98 (0.092)	-1.01 (0.225)	-0.01 (1.00)			
$50,000 - $79,999 (C)	12.43				-0.04 (1.00)	0.96 (0.058)			
$80,000 - $99,999 (D)	12.47					1.00 (0.132)			
$100,000 or greater (E)	11.47								
**Workstatus^**		A	B						
No (A)	11.87		0.68 (0.025)						
Yes (B)	11.12								

Based on estimated marginal means

Adjustment for multiple comparisons: Bonferroni

## Discussion

The dietary supplement (DS) industry is growing, and the cost burden of DS on older adults is substantial. Similar to prior studies which have shown that approximately 75% of older adults use DS [7], the percentage of participants who use DS ranged from 70.4 to 82.7% from baseline to year 5. This translates to a considerable cost burden for those who use DS, despite conflicting or absent data regarding the health benefits of DS. Our analysis showcases that the mean annual cost of DS in this population is $186 as a conservative estimate. As healthcare costs rise, increased attention is needed to better understand relative DS costs among older adults, as well as the impact of those costs, which could include prescription medication nonadherence, food insecurity, and many other financially sensitive determinants of health.

Previously published data from the AAA LongROAD study noted that older adults tend to use a variety of supplements that often overlap in their ingredients and can lead to redundant use. In addition to increasing the pill burden and the potential for inadvertent consumption of excess doses of DS, DS redundancy can increase the cost burden.

There was a wide range of estimated DS costs per participant, from $0.73 to $174.80 monthly. A few participants spent far more than the remaining participants, and these high DS utilizers are a group of interest for future analysis. The mean annual estimated cost burden of DS among DS users in this analysis was $186, lower than that in a 2012 cost analysis which estimated the mean out of pocket annual expenditure for natural product supplements to be $369 per adult [20]. This difference may be explained by different methods of estimating cost and illustrates the difficulty in assessing real-world cost burden. It is also possible that with the increase in DS industry size, DS prices have decreased, thus offsetting the increase in DS utilization and resulting in a lower or comparable total annual cost per individual. Nevertheless, this amount represents approximately 5% of the out of pocket medical spending among traditional Medicare beneficiaries [21].

There was also a wide range of costs for individual DS, from $0.73 to $48.59. As trends in DS use change, increased or decreased costs of specific supplements may drive market-level changes in consumer spending, as well as individual-level cost burdens for older adults. Those who spent more on DS were more likely to be female, White non-Hispanic, educated, and in the lowest income bracket. Regional differences, such as greater spending at the Denver CO site and lower spending at Baltimore MD, should be further explored. Given the abundance of online options for shopping and price comparisons, these regional differences are less likely to be from price variability, and more likely due to demographic factors and/or the culture of DS utilization.

Future analyses will need to further explore the relationship between the DS cost burden and clinical measures and outcomes, and assist older adults in making medically-sound decisions about medication and DS use. For example, prior studies have shown that the cost of prescription medications is a primary factor in nonadherence [22], while at the same time, older adults are also increasing their DS use and spending more on DS [6]. It remains unclear whether DS use has an impact on prescription medication adherence, such as substituting more expensive prescription medication with less expensive DS. To date, studies examining the relationship between DS use and medication adherence are limited and mixed, with some studies showing medication discontinuation or nonadherence among DS users [23, 24], and others showing no impact at all [25, 26].

To our knowledge, there have been no prior studies evaluating the DS cost burden in a large, multicenter longitudinal cohort. A strength of this analysis is the use of a brown bag medication review instead of chart review or patient recall, providing a more comprehensive catalog of an individual’s total medications. By using data from a large, multicenter cohort, some geographical variations in DS use are appreciated, although all study sites are affiliated with large medical centers.

Limitations of the use of this cohort of older drivers include a trend toward higher income and White non-Hispanic population, compared with the general U.S. population of older adults. While loss to follow up may further impact DS utilization rates and associated estimated costs, this 5-year longitudinal review allows for the estimation of cost burden over time. In terms of the accuracy of measuring DS use and cost estimation, DS use may be overestimated by relying on once yearly review extrapolated to yearlong use. However, the overall number of supplements in each time period was somewhat stable, indicating that participants at least substitute, if not continue the same, DS. As such, the annual cost burden would remain similar. Additionally, by using a lower-cost supplier to estimate DS prices, cost burdens are underestimated; comparing the selected supplier to multiple other retailers increases confidence in the underestimation of cost. Since DS costs at the time of each study year were not obtained, costs from 2022 were extrapolated and may result in over- or underestimated on the basis of fluctuations in market values. Finally, the exclusion of certain DS (topical, other, undetermined) from the cost analysis again results in an underestimate of an individual’s total cost burden. While it is possible that these excluded items were systematically over- or underused in one population group or another, they represented a minority of the total DS and their exclusion was unlikely to change significant relationships.

## Conclusions

Many older adults have fixed incomes, and the relative costs of DS remain poorly understood. Using a lower-priced online retailer to create conservative cost estimates, the cost burden of DS per older adult remains high. Those who identified as female and White non-Hispanic spent significantly more on DS, while there was no significant difference by education or income level. Further research should detail real-world DS costs and evaluate the impact of DS costs among older adults.

## Data Availability

Data used in this study may be available from the corresponding author upon reasonable request and with permission of the AAA Foundation for Traffic Safety.

## Abbreviations

DS: Dietary supplements
LongROAD: Longitudinal Research on Aging Drivers
AHFS: American Hospital Foundation Service
SD: Standard deviation
